# Hyperspectral Leaf Reflectance as Proxy for Photosynthetic Capacities: An Ensemble Approach Based on Multiple Machine Learning Algorithms

**DOI:** 10.3389/fpls.2019.00730

**Published:** 2019-06-03

**Authors:** Peng Fu, Katherine Meacham-Hensold, Kaiyu Guan, Carl J. Bernacchi

**Affiliations:** ^1^Department of Plant Biology, University of Illinois at Urbana-Champaign, Urbana, IL, United States; ^2^Carl R. Woese Institute for Genomic Biology, University of Illinois at Urbana-Champaign, Urbana, IL, United States; ^3^National Center for Supercomputing Applications, University of Illinois at Urbana-Champaign, Urbana, IL, United States; ^4^Department of Natural Resources and Environmental Sciences, University of Illinois at Urbana-Champaign, Urbana, IL, United States; ^5^USDA-ARS Global Change and Photosynthesis Research Unit, University of Illinois at Urbana-Champaign, Urbana, IL, United States

**Keywords:** photosynthesis, high-throughput phenotyping, machine learning, stacked regression, gas exchange system

## Abstract

Global agriculture production is challenged by increasing demands from rising population and a changing climate, which may be alleviated through development of genetically improved crop cultivars. Research into increasing photosynthetic energy conversion efficiency has proposed many strategies to improve production but have yet to yield real-world solutions, largely because of a phenotyping bottleneck. Partial least squares regression (PLSR) is a statistical technique that is increasingly used to relate hyperspectral reflectance to key photosynthetic capacities associated with carbon uptake (maximum carboxylation rate of Rubisco, *V_c,max_*) and conversion of light energy (maximum electron transport rate supporting RuBP regeneration, *J_max_*) to alleviate this bottleneck. However, its performance varies significantly across different plant species, regions, and growth environments. Thus, to cope with the heterogeneous performances of PLSR, this study aims to develop a new approach to estimate photosynthetic capacities. A framework was developed that combines six machine learning algorithms, including artificial neural network (ANN), support vector machine (SVM), least absolute shrinkage and selection operator (LASSO), random forest (RF), Gaussian process (GP), and PLSR to optimize high-throughput analysis of the two photosynthetic variables. Six tobacco genotypes, including both transgenic and wild-type lines, with a range of photosynthetic capacities were used to test the framework. Leaf reflectance spectra were measured from 400 to 2500 nm using a high-spectral-resolution spectroradiometer. Corresponding photosynthesis vs. intercellular CO_2_ concentration response curves were measured for each leaf using a leaf gas-exchange system. Results suggested that the mean *R*^2^ value of the six regression techniques for predicting *V_c,max_* (*J_max_*) ranged from 0.60 (0.45) to 0.65 (0.56) with the mean *RMSE* value varying from 47.1 (40.1) to 54.0 (44.7) μmol m^-2^ s^-1^. Regression stacking for *V_c,max_* (*J_max_*) performed better than the individual regression techniques with increases in *R*^2^ of 0.1 (0.08) and decreases in *RMSE* by 4.1 (6.6) μmol m^-2^ s^-1^, equal to 8% (15%) reduction in *RMSE*. Better predictive performance of the regression stacking is likely attributed to the varying coefficients (or weights) in the level-2 model (the LASSO model) and the diverse ability of each individual regression technique to utilize spectral information for the best modeling performance. Further refinements can be made to apply this stacked regression technique to other plant phenotypic traits.

## Introduction

Increasing demands for food, fiber, and fuel caused by rising human population and global affluence will be a burden to environment sustainability over the next several decades. These increasing demands are likely to be challenged further with the world’s shrinking farmlands (Sayer et al., 2013; Ort et al., 2015) and with climate change (Tester and Langridge, 2010). Among other improvements, development of high photosynthetically efficient crop cultivars is required to overcome these challenges (Tester and Langridge, 2010). Although crop yields have increased over the last several decades, this is achieved in the Green Revolution which are diminishing with time (Parry et al., 2011). Photosynthesis as a process leaves significant room for improvement, which can bolster crop yields (Long et al., 2006; Zhu et al., 2008). Thus, major research efforts are underway to increase photosynthetic energy conversion efficiency by engineering photosynthetic pathways (Yokota and Shigeoka, 2008; Ducat and Silver, 2012; Ort et al., 2015) and exploiting mechanisms underlying natural variation of photosynthesis (Flood et al., 2011; Lawson et al., 2012).

Altering the photosynthetic capacity of plants may lead to higher productivity, but assessing the potential to optimize photosynthesis, or to measure the underlying natural variation in multiple plots representing diverse genotypes requires careful and comprehensive phenotyping under field conditions (Furbank and Tester, 2011). High-throughput phenotyping using non-invasive imaging sensors offers a non-destructive, rapid, and inexpensive way to characterize phenotypic traits for individual plants (Finkel, 2009; Großkinsky et al., 2015). However, compared with high-throughput genotyping (Thomson, 2014), plant phenotyping in a low-throughput manner has been a bottleneck to the generation of improved crop varieties (Furbank and Tester, 2011). Therefore, advances in both high-throughput phenotyping platforms (HTPPs) and statistical techniques that relate sensor measurements to phenotypic traits are needed to enable capacity for rapid and accurate phenotyping to ensure crop improvements.

Biochemical kinetic properties such as *V_c,max_* (the maximum rate of Rubisco-catalyzed carboxylation) and *J_max_* (maximum electron transport rate supporting RuBP regeneration) are critical variables in determining photosynthetic capacity (Long and Bernacchi, 2003). These parameters with their underlying temperature functions (Bernacchi et al., 2001, 2003) are used to parameterize the leaf photosynthesis model (Farquhar et al., 1980) to predict photosynthetic rates over a wide range of environmental conditions. Traditionally, these parameters are acquired from *in vivo* measurements using commercial gas exchange systems (Long and Bernacchi, 2003) fit to mechanistically defined photosynthesis models (Farquhar et al., 1980; Sharkey et al., 2007). However, measurements from gas exchange systems are time-consuming, cost-prohibitive, and labor-intensive, making it difficult to phenotype photosynthesis for large numbers of plants in a short time. The emergence of HTPPs in recent years suggests opportunities to rapidly measure leaf level photosynthetic information for thousands of individual plants. Imaging techniques currently used in HTPPs include visible light (RGB), fluorescence, thermal, 3D (e.g., light detection and ranging), tomographic, and hyperspectral imaging (HSI) (Fiorani et al., 2012; Deery et al., 2014; Li et al., 2014). Among these techniques, HSI is deemed as one of the most effective technologies to predict physiological status and stress related response of crops in a high-throughput manner at different scales (Mahlein et al., 2012; Matsuda et al., 2012; Mutka and Bart, 2014; Sytar et al., 2017).

Inference of photosynthetic variables and other phenotypic traits from hyperspectral reflectance entails the development of calibration models relating spectral measurements and reference data (e.g., *V_c,max_* and *J_max_*, derived with gas exchange systems). Required by model calibrations, a representative sub-sample of a complete data set in terms of range of spectral variation treated with appropriate pre-processing techniques should be selected (Montes et al., 2007; Cabrera-Bosquet et al., 2012). In model calibration phase, empirical models used to correlate spectral information with ground truth data can be diverse. For most HSI studies, vegetation indices that associate two or more spectral bands with specific biological parameters of plants/crops are commonly derived for assessing and quantifying phenotypic traits (Fiorani et al., 2012). As such, simple correlation, regression, and classification techniques rather than sophisticated mathematical models can help achieve research goals, for example to characterize plant responses to abiotic and biotic factors (Rumpf et al., 2010; Kim et al., 2011; Behmann et al., 2014). In contrast, to relate photosynthetic capacities with complete reflectance spectra, it is necessary to use statistical models that have both powerful feature extraction ability and data inference ability. For example, partial least squares regression (PLSR) (Geladi and Kowalski, 1986; Wold et al., 2001) has been commonly used to estimate *V_c,max_* and *J_max_* at the leaf level from leaf-clip reflectance spectra (Serbin et al., 2012; Ainsworth et al., 2014; Yendrek et al., 2017; Silva-Perez et al., 2018). These studies also showed that wavebands used for estimating photosynthetic information fell with spectral regions associated with leaf characteristics such as water content, internal structure, dry mass, and chlorophylls. However, the performance of PLSR in estimating photosynthetic capacities varies significantly across different plant species, regions, and growth environments.

To cope with the heterogeneous performances of PLSR among different situations, it is necessary to explore other powerful machine learning techniques. With appropriate feature extraction, other statistical techniques such as artificial neural network (ANN) regression (Specht, 1991), support vector machine (SVM) regression (Cortes and Vapnik, 1995), least absolute shrinkage and selection operator (LASSO) regression (Tibshirani, 1996), random forest (RF) regression (Breiman, 2001), and Gaussian process (GP) regression (Williams and Rasmussen, 2006) may achieve the similar, if not better, predictive performance as PLSR in phenotyping photosynthetic variables. However, there is a lack of understanding of the predictive performance of individual machine learning-based regression techniques and whether their ensemble would provide better performances for quantifying photosynthetic variables in a high-throughput manner. Therefore, the objectives of this study are to test a series of regression techniques, including PLSR, and compare the model performance of each individual regression technique to that of stacking all the regression techniques in high-throughput phenotyping photosynthetic capacities. Testing these machine learning techniques on both wild and genetically modified tobacco plants, we hypothesize that this stacked regression framework may form a more general approach to estimations of plant phenotypic traits of greater accuracy and sensitivity than those from any single regression algorithm.

## Materials and Methods

### Experimental Site

Six tobacco (*Nicotiana tabacum*) genotypes including both transgenic and wild type lines (Table 1) were planted during two growing seasons (2016–2017) at the University of Illinois Energy Farm Facility in Urbana, Illinois^1^. Tobacco plants were germinated in green house conditions and transplanted to the farm field at the four leaf stage. Two weeks prior to transplanting, 275 lbs./acre ESN Smart Nitrogen (∼150 ppm) was applied to the field site. A biological pesticide, *Bacillus thuringiensis v. kurstaki* (54%) (DiPel PRO, Valent BioSciences LLC, Walnut Creek, CA, United States), was applied to the field site 5 days prior to transplanting and bi-weekly thereafter to control for tobacco pests. In addition, a broad action herbicide, Glyphosate-isopropylammonium (41%) (Killzall; VPG, Windthorst, TX, United States) was applied once to all plots 2 days before transplanting at 15 l at 70 g/l. Each genotype plot was arranged in a 6 plant × 6 plant grid totaling 36 plants per plot with 0.38 m spacing and was replicated four times. Throughout the growing season, irrigation was provided to tobacco plants as needed. The six genotypes have quite contrasting differences in photosynthetic capacities with three wild-type cultivars of different growth rates, two transgenic Rubisco antisense lines with reduced photosynthetic capacity (Hudson et al., 1992), and one transgenic type with overexpression of photosynthetic carbon reduction cycle enzymes to increased photosynthetic capacity (Simkin et al., 2015; Table 1). Thus, these genotypes can provide a wide range for each photosynthetic variable. In this study, photosynthetic capacities *V_c,max_* and *J_max_* (ambient values rather than values normalized to a standard temperature) were derived, as described below.

**Table 1:** List and description of tobacco genotypes used in the study.

Genotype	Transgene	Transgene expected function
Petit Havana	None, wild type	n/a
Samsun	None, wild type	n/a
Mammoth	None, wild type	n/a
SFX	Overexpressed photosynthetic carbon reduction cycle enzymes. Background: Samsun	Improved photosynthetic capacity, due to increased carbon reduction enzymes
Single Rubisco Knockdown (SSuS)	Rubisco small subunit antisense. 40% of WT Rubisco. Background: W38	Reduced photosynthetic capacity, due to reduced Rubisco activity
Double Rubisco Knockdown (SSuD)	Rubisco small subunit antisense. 10% of WT Rubisco. Background: W38	Reduced photosynthetic capacity, due to reduced Rubisco activity


### Leaf Reflectance and Gas Exchange

Leaf reflectance properties of the six genotypes were analyzed from 400 to 2500 nm using a high-spectral-resolution spectroradiometer (Fieldspec 4, Analytical Spectral Devices – ASD, Boulder, CO, United States) with a leaf clip attached to the fiber optic cable. The spectroradiometer has a spectral resolution of 3 nm in the visible and near infrared range (350–1000 nm) and of 8 nm in the shortwave-infrared range (1000–2500 nm). The relative leaf reflectance was determined from the measurement of leaf radiance divided by the radiance of a 99% reflective white standard (Spectralon, Labsphere Inc., North Dutton, NH, United States). Six leaf-clip reflectance measurements were made in different regions of the same youngest fully expanded sunlit leaf and then were averaged. Measurements were collected between 11 AM and 2:30 PM local time under clear-sky conditions for three different leaves in each plot. The short-time window was to ensure that photosynthetic variation among cultivars in a day were not impacted by time. Within 30 min of hyperspectral measurements, the corresponding response of photosynthesis (*A*) to intercellular CO_2_ concentration (*C_i_*) for each leaf was captured using a portable leaf gas exchange system (LI-6400, LICOR Biosciences, Lincoln, NE, United States). Measurements were initiated at the growth CO_2_ concentration of 400 μmol m^-2^ s^-1^ at saturating light (1800 μmol m^-2^ s^-1^). The CO_2_ concentration (μmol mol^-1^) in the cuvette was changed stepwise in the following order: 400, 200, 50, 100, 300, 400, 600, 900, 1200, 1500, 1800, and 2000. Prior to initiating *A/C_i_* curves, three leaf temperature measurements were made and averaged using a handheld IR temperature probe (FLIR TG54, FLIR Systems, Inc., Wilsonville, OR, United States) and the block temperature of the gas exchange cuvette was set to match this average leaf temperature. In addition, leaves were acclimated to chamber conditions for a minimum of 300 s and adjusted to chamber conditions for between 160 s and 200 s before each individual measurement. Relative humidity inside the chamber was controlled at 65 ± 5% by adjusting the flow through the desiccant tube integrated into the gas exchange system. The photosynthetic variables *V_c,max_* and *J_max_* were derived by fitting *A/C_i_* curves with a mechanistically defined mathematical model (Farquhar et al., 1980) through a fitting utility program (Sharkey et al., 2007). The mesophyll conductance (*g_m_*) was constrained according to a previous study for tobacco at 25°C (Evans and Von Caemmerer, 2012). According to Sharkey (2016), the derived *J_max_* should be called as *J* or *J* at 1800 μmol m^-2^ s^-1^ and should not be used for the maximum rate of electron transport at high light intensity. Thus, in the following of the manuscript, we used *J*_1800_ instead of *J_max_* when referring to both measured and predicted values.

The pairs of reflectance spectra and *A/C_i_* curves were measured on the following dates from 2016 to 2017: June 30-July 1, 2016, July 19 and 21, 2016, August 4 and 5, 2016, June 22 and 28 2017, July 6, 7, 12 and 31 2017, and August 1 and 18 2017. In total, 212 data pairs were collected for *V_cmax_*, and 179 measurement pairs for *J*_1800_. The fewer measurement pairs of *J*_1800_ than *V_cmax_* stems from the double Rubisco knockdown plants (SSuD) not being electron transport limited under any conditions and therefore were removed from analysis. Further details can also be found in Meacham-Hensold et al. (2019).

## Regression Techniques

**FIGURE 1:**
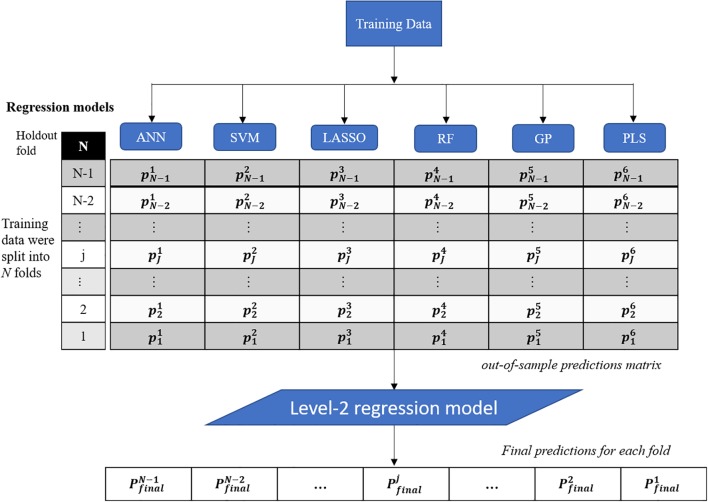
The workflows of regression stacking for phenotyping photosynthetic capacities. ANN, artificial neural network; SVM, support vector machine; LASSO, least absolute shrinkage and selection operator; RF, random forest; GP, Gaussian process; and PLS, partial least squares. *P* and *p* are model predictions at different modeling stage. The regression models are trained with a leave-one-out cross validation approach (the *Nth* fold is reserved) to form the out-of-sample predictions matrix. The final predictions of each fold were made using the LASSO model based on the out-of-sample predictions matrix (no data normalization).

This study presents a test of the idea that an ensemble of regression techniques can be used together to measure plant traits with greater accuracy and sensitivity than from any single regression algorithm. Stacked regression (SR, also called as stacked generalization, stacking, stacking regressions, or blending) was first introduced by Wolpert (1992) and later statistically principled by Breiman (1996) to blend different predictors to give improved prediction accuracy. Although SR is used less frequently than other ensemble learning methods, such as Bagging and Boosting, it is commonly used for generating ensembles of heterogeneous predictors (Sesmero et al., 2015). Figure 1 shows the workflows of stacked regression for phenotyping photosynthetic capacities. The training data pairs, leaf level hyperspectral reflectance and gas exchange system-derived *V_c,max_* and *J*_1800_ were first split into *N* folds (*N* was 10 in this study) with the *N*th fold reserved for test. In this study, six regression models including ANN, SVM, LASSO, RF, GP, and partial least squares (PLS) regressions were individually tested and combined in the stacked regression framework. As seen from Figure 1, predictions for each fold were obtained using the *N-2* folds and collected in an out-of-sample predictions matrix. Then the out-of-sample predictions matrix was used to train the level-2 regression model to obtain final predictions for all data points. Here the LASSO regression was used as the level-2 model to avoid collinearity among predictions of photosynthetic capacities. To reduce uncertainty, the 10-fold cross-validations were conducted for both level-1 and level-2 models. More importantly, by using the cross-validated predictions, SR avoids giving unfair weight to models with higher complexity. In this study, the data pairs collected in 2016 and 2017 were randomly split into the training and test datasets with a ratio of 9:1. This splitting procedure was repeated 10 times for analysis of the performance of both the six regression techniques and the stacked regression.

**FIGURE 2:**
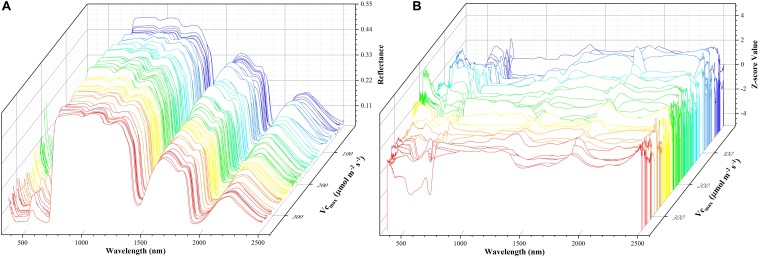
The spectra data: a matrix of 212 samples (rows) and 2151 features (columns). The *x*-axis refers to the wavelength, the *y*-axis represents *V_c,max_*, and the *z*-axis denotes the reflectance of the spectrum **(A)** or *z*-scored value **(B)**. Each color line represents one sample.

Before the training of each individual regression model, the original hyperspectral reflectance data of samples were standardized for each individual band as:

$$z=\frac{R_{i}-\overset{¯}{R_{i}}}{S_{R_{i}}}$$

where *z* refers to the standardized reflectance value, *R_i_* is the raw hyperspectral reflectance for band *i*, *R_i_* is the mean value of all the sampled hyperspectral reflectance for band *i*, and *S_R_i__* is the standard deviation of all the sampled hyperspectral reflectance for band *i*. This pre-processing step ensures that reflectance values at each wavelength have zero mean and unit standard deviation and receive equal considerations in the model training phase. For the level-2 model, the out-of-sample predictions (without data normalization) were directly used for regression stacking. Figure 2 shows the raw spectra and standardized data in 3D. During the model training and test phases, the performance of each individual model and stacked regression was assessed based on the coefficient of determination (*R*^2^) and root mean square error (*RMSE*). In the following sections, a brief overview of each individual regression technique was provided.

### Artificial Neural Network

Artificial neural network models are generic non-linear function approximation algorithms that are capable of computing, predicting, and classifying data (Ali et al., 2016). They have been widely used in applications including pattern recognition, classification, and regression in various fields (Hong et al., 2004; Kim, 2010; Zain et al., 2012; Neto et al., 2017). ANN refers to a multi-layer network structure that consists of an input layer, an output layer, and one or more hidden layers (Kimes et al., 1998). It achieves regression by building a model of the data-generating process for the network to generalize and predict outputs from inputs that are not previously seen. In this study, back-prorogation (BP) neural networks-based regression were utilized in that it can handle non-linear relationships among data even when there are conflicting relationships between the input variables and the response variables (Moghadassi et al., 2010). The optimal number of hidden layers and neurons in the BP neural networks was determined through the leave-one-out cross validation process that yielded the smallest *RMSE* value.

### Support Vector Machine

Support vector machine, benefiting from the statistical learning theory and the minimum structural risk principle (Cortes and Vapnik, 1995), is mainly used for classification and regression of small non-linear and high-dimensional samples (Mountrakis et al., 2011). Given a set of adequate training samples, support vector regression (SVR) allows continuous estimations of a specified output variable by fitting an optimal approximating hyperplane to a set of training samples. Such a hyperplane is approximated with two important parameters including the kernel function, which reflects similarity between data points (i.e., between reflectance values), and the cost loss function (regularization parameter; Verrelst et al., 2012). Integrated into a kernel framework, SVR enables mapping the original data into a higher dimensional feature space, wherein a better fitting of a linear function would be possible (Brereton and Lloyd, 2010). In this study, the radial basis function (RBF) was used as the kernel function with the regularization parameter tuned through the cross-validation process.

### Least Absolute Shrinkage and Selection Operator

Least absolute shrinkage and selection operator as a regression analysis method performs both variable selection and regularization by minimizing the residual sum of squares subject to the sum of the absolute value of the coefficients being less than a constant (Tibshirani, 1996). It was originally introduced in the context of least squares as a sparse regression method. In building a model with high dimensional data such as hyperspectral reflectance, LASSO regression can shrink some of the regression coefficients toward zero as the penalty parameter increases to improve the prediction accuracy (Donoho, 2006). As a quadratic programming problem, LASSO regression coefficients can be optimized and derived by efficient algorithms without much computational cost (Efron et al., 2004; Friedman et al., 2010; Boyd et al., 2011). In this study, the LASSO regression was utilized since it has been widely used to deal with hyperspectral data for various purposes (Samarov et al., 2015; Sara et al., 2017; Yang and Bao, 2017).

### Random Forest

Random forest is a non-linear statistical ensemble method that constructs and subsequently averages a large number of randomized decision trees for classification or regression (Breiman, 2001). It models the relationship between explanatory variables and response variables by a set of decision rules which are constructed by recursively partitioning the input space into successively smaller regions (Hastie et al., 2009). RFs overcome weaknesses of regression trees that tend to overfit the data as the tree becomes too complex (James et al., 2013) by introducing randomness through a bootstrap strategy. Generally, the number of variables selected at each split tree was optimized by minimizing the out-of-bag error of predictions. In this study, RF regression was selected because it can handle data of high dimensions and does not require explicitly the feature selection step (Hastie et al., 2009).

### Gaussian Process

The Gaussian process regression (GPR) can be interpreted as a distribution over and inference occurring in the space of function from the function-space view (Williams and Rasmussen, 2006). It has been received much attention in the field of machine learning and can provide the Bayesian approach to establishing the relationship between the input (i.e., hyperspectral reflectance) and the output variable. GPR achieves the prediction purpose by computing the posterior distribution over the unknown values with the hyperparameters typically tuned by maximizing the Type-II Maximum Likelihood, using the marginal likelihood of the observations. In this study, GPR was employed since it has been widely used for remote sensing applications (Verrelst et al., 2013; Fu and Weng, 2016).

### Partial Least-Squares Regression

Partial least square regression (PLSR) is a bilinear calibration method using data reduction by compressing a large number of measured collinear variables into a few orthogonal principal components (also known as latent variables) (Geladi and Kowalski, 1986; Wold et al., 2001). These latent variables represent the main variance-covariance structures as they are constructed to optimize the explained power of the response variables (Ehsani et al., 1999). PLSR estimates the regression coefficients for each latent variable through a leave-one-out cross validation approach. In general, the optimal number of latent variables is determined by minimizing *RMSE* between predicted and observed response variable. More details on the PLSR algorithm can be referred to Esbensen et al. (2002).

## Results

### The Modeling Performance for Predicting *V*_c,max_ and *J*_1800_

**FIGURE 3:**
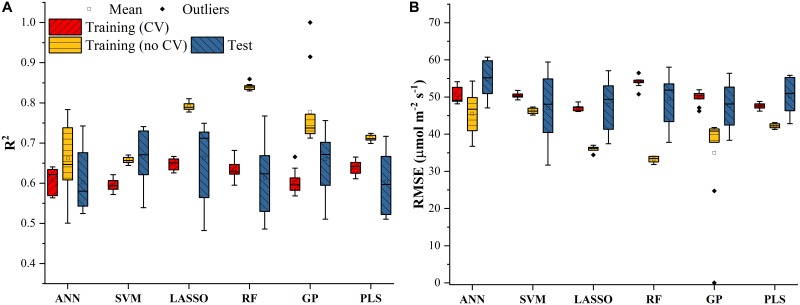
The statistical distributions of *R*^2^ **(A)** and *RMSE* **(B)** of each machine learning algorithm for predicting *V_c,max_* in the training (with and without cross-validation) and test phases. The training phase with cross-validation was required by the regression stacking, and models trained without cross-validation were used in the test phase.

The *V_c,max_* and *J*_1800_ datasets collected based on the leaf gas exchange systems in 2016 and 2017 exhibited a variation of 23.8 fold (14.5-344.6 μmol m^-2^ s^-1^) and 4.9 fold (73.6-362.0 μmol m^-2^ s^-1^), respectively (Table 2). The six cultivars had quite varying mean and standard deviation values for *V_c,max_* and *J*_1800_. Figure 3 shows the statistical distributions of *R*^2^ and *RMSE* values of each machine learning algorithms for predicting *V_c,max_*. With the cross-validation in the model training phase, the LASSO model yielded the highest mean *R*^2^ of 0.65 (mean *RMSE* = 47.1 μmol m^-2^ s^-1^), followed by the PLS model with the mean *R*^2^ of 0.64 (mean *RMSE* = 47.6 μmol m^-2^ s^-1^). The SVM regression and the GP regression had the same mean *R*^2^ value of 0.60 with the *RMSE* value of 50.4 μmol m^-2^ s^-1^ and 49.8 μmol m^-2^ s^-1^, respectively. Compared to the ANN regression model (mean *R*^2^ = 0.61; mean *RMSE* = 50.5 μmol m^-2^ s^-1^), the RF model had a higher mean *R*^2^ of 0.63 with a larger mean *RMSE* of 54.0 μmol m^-2^ s^-1^. Among the six regression models, LASSO displayed the smallest standard deviation in both *R*^2^ and *RMSE* while the largest standard deviation was found in ANN for both *R*^2^ and *RMSE*. In the model test phase, it was found that the *R*^2^ and *RMSE* values of each regression model had a relatively wider range, compared to those in the model training phase. For example, the *R*^2^ and *RMSE* yielded by the LASSO model in the model test phase ranged from 0.48 to 0.75, much wider than the range from 0.62 to 0.7 in the model training phase. In addition, the mean *R*^2^ and *RMSE* values of each machine learning algorithm were slightly larger or at least very similar to those in the model training phase. These findings were reasonable since the machine learning algorithms applied to test dataset were calibrated by all the training data rather than data used in the cross-validation in the training phase (Figure 3). The best regression model (based on the mean *R*^2^ and *RMSE* values) in the model test phase was achieved by SVM (*R*^2^ = 0.67, *RMSE* = 47.1 μmol m^-2^ s^-1^), followed by GP (*R*^2^ = 0.66, *RMSE* = 47.7 μmol m^-2^ s^-1^), LASSO (*R*^2^ = 0.66, *RMSE* = 47.9 μmol m^-2^ s^-1^), RF (*R*^2^ = 0.61, *RMSE* = 49.5 μmol m^-2^ s^-1^), PLS (*R*^2^ = 0.60, *RMSE* = 50.1 μmol m^-2^ s^-1^), and ANN (*R*^2^ = 0.60, *RMSE* = 54.8 μmol m^-2^ s^-1^). The disparities of the performance of different regression models, when applied to the same dataset, further suggested that it was necessary to develop new techniques to utilize the advantages but avoid the disadvantages of each individual regression algorithm.

**Table 2:** The descriptive statistics of *V_c,max_* (μmol m^-2^ s^-1^) and *J*_1800_ (μmol m^-2^ s^-1^) for samples collected in 2016 and 2017 from the energy farm at University of Illinois at Urbana-Champaign.

Genotype	Sample number	V*_c,max_* Range	V*_c,max_* ± Std Dev	Jmax Range	Jmax ± Std Dev
Petit Havana	38	80.3 - 238.1	166.0 ± 38.2	156.8 - 290.4	230.5 ± 27.9
Samsun	39	139.9 - 344.6	239.5 ± 61.8	169.4 - 362.0	267.8 ± 51.4
Mammoth	39	89.0 - 339.9	208.1 ± 65.1	92.8 - 342.4	233.4 ± 62.7
SFX	18	218.1 - 271.1	244.7 ± 15.6	252.5 - 323.2	285.0 ± 16.0
SSuS	45	29.9 - 241.5	160.6 ± 55.3	73.6 - 330.7	227.9 ± 64.6
SSuD	33	14.5 - 166.2	55.3 ± 37.9	N/A	N/A
*Overall*	212 (179)	14.5 - 344.6	175.5 ± 79.2	73.6 - 362.0	243.9 ± 55.5


*The range and mean values ( ± standard deviation) of *V_*c,max*_* and *J_1800_* are provided for each tobacco cultivar as well as for the whole dataset based on leaf gas exchange measurements. In total, 212 data samples were collected for *V_*c,max*_*, and 179 samples for *J*_1800_.*

**FIGURE 4:**
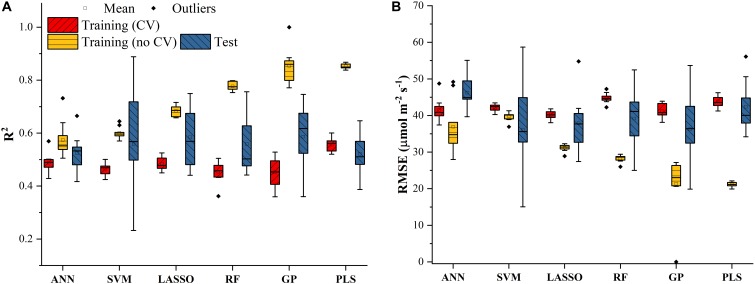
The statistical distributions of *R*^2^ **(A)** and *RMSE* **(B)** of each machine learning algorithm for predicting *J*_1800_ in the training (with and without cross-validation) and test phases. The training phase with cross-validation was required by the regression stacking, and models trained without cross-validation were used in the test phase.

Similar results were also found in Figure 4 for predicting *J*_1800_ using the six regression models. However, compared to the mean *R*^2^ values of the regression models in Figure 3, those in Figure 4 were much smaller and were generally less than 0.6 in the model training phase. The best predictive performance was achieved by the PLSR with the mean *R*^2^ value of 0.56 and a *RMSE* value of 43.8 μmol m^-2^ s^-1^. Compared to PLSR, the LASSO model had a smaller mean *R*^2^ value of 0.48 with a smaller mean *RMSE* value of 40.1 μmol m^-2^ s^-1^. It was also noted that the ANN model (*R*^2^ = 0.48, *RMSE* = 41.5 μmol m^-2^ s^-1^) exhibited a similar predictive performance to the LASSO model but with a relatively narrower *R*^2^ range. The SVM, RF, and GP had a very similar predictive performance, with the GP model exhibiting the largest standard deviation values in both *R*^2^ and *RMSE*. Higher mean *R*^2^ values and smaller *RMSE* values were observed in the model test phases compared to those in the training phase. The improved performance is likely attributed to the better trained machine learning algorithms using all the samples as the training data (Figure 4). Overall, the differences among the performance of each individual regression model in predicting *V_c,max_* and *J*_1800_ across different cultivars over time provided a strong basis for stacking (see section “The Regression Stacking”).

### The Regression Stacking

**FIGURE 5:**
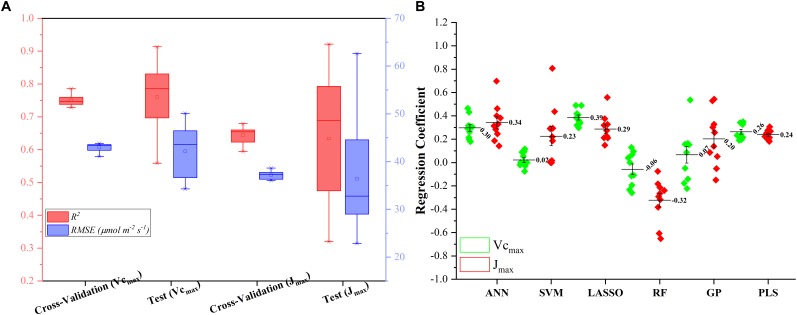
The statistical distributions of *R*^2^ and *RMSE* of the regression stacking for predicting *V_c,max_* and *J*_1800_ in the training (cross-validation) and test phases **(A)**, and the distribution of coefficient within the level-2 model (LASSO) **(B)**.

Figure 5A presents the modeling performance of the regression stacking (the LASSO model as the level-2 model as shown in Figure 1) for predicting *V_c,max_* and *J*_1800_ in both the training and test phases. For *V_c,max_*, the regression stacking improved the mean *R*^2^ value to 0.75, an increase of 0.1 compared to the highest mean *R*^2^ value observed in the LASSO model (Figure 3) in the training phase (cross-validation). Meanwhile, the mean *RMSE* value in the regression stacking was reduced to 43.0 μmol m^-2^ s^-1^, less than the mean *RMSE* value of 47.1 μmol m^-2^ s^-1^ yielded by the LASSO model. Still, a slightly higher mean *R*^2^ value of 0.76 and a smaller mean *RMSE* value of 42.2 μmol m^-2^ s^-1^ were observed in the test phases compared to those in the training phase. Similar findings were also observed in the performance of the stacking regression to predict the *J*_1800_ parameter (Figure 5A) in the training and test phases. For *J*_1800_, the stacking regression yielded the mean *R*^2^ value of 0.64 and the mean *RMSE* value of 37.2 μmol m^-2^ s^-1^ in the training phase. An increase of 0.08 in the *R*^2^ value and a decrease of 6.6 μmol m^-2^ s^-1^ in the *RMSE* value were noted in the training phase of the stacking regression compared to the best model (the PLS model with the *R*^2^ of 0.56 and the *RMSE* value of 43.8 μmol m^-2^ s^-1^) used to predict the *J*_1800_ parameter (Figure 4). In the test phase, the stacked regression provided a higher *R*^2^ value of 0.63 and a *RMSE* value of 36.4 μmol m^-2^ s^-1^. It was also worth noting that the mean *R*^2^ and *RMSE* values yielded by the regression stacking in the training phase were very similar to those in the test phase for predicting both *V_c,max_* and *J*_1800_. Overall, the performance improvement of the regression stacking should be credited to the ability of the regression stacking to harness strengths of each individual model.

To further explain the mechanism of the better performance of the regression stacking, Figure 5B shows the distribution of the coefficient of each individual regression model within the level-2 model (the LASSO model). A larger coefficient within the level-2 model indicated a higher weight in the stacking procedure. As shown in Figure 5B, the stacking performance depended heavily on the LASSO, ANN, and PLS models with the mean coefficients (standard deviation) of 0.39 (0.07), 0.30 (0.09), and 0.26 (0.07), respectively, for predicting the *V_c,max_* parameter. The impacts of the SVM, RF and GP models on the stacking were relatively small even though the standard deviation values of their coefficients could reach up to 0.22. The larger standard deviation values of the SVM, RF, and GP model, compared to the other three models, may indicate that the three models were more sensitive to the changes in the training dataset. These results suggest that the sampling strategy should be optimized to generate a representative training dataset to feed into the SVM and RF models for a good modeling performance.

For the prediction of *J*_1800_, the distribution of the coefficient of each regression model was quite different from that in the prediction of *J*_1800_. The highest coefficient was found in the ANN model (0.34 ± 0.15), followed by the LASSO (0.29 ± 0.11), PLS (0.24 ± 0.04), SVM (0.23 ± 0.25), GP (0.20 ± 0.23), and RF (-0.32 ± 0.23) models. Still, the coefficient of the SVM, RF, and GP within the level-2 model, compared to that in other three models, displayed relatively higher standard deviation though the RF model was negatively used in the stacking for predicting *J*_1800_. Overall, these findings indicated that the stacking procedure was better than each individual regression techniques for predicting photosynthetic capacities.

## Discussion

### Explanations for Heterogeneous Modeling Performance of Machine Learning Algorithms to Predict *V*_c,max_ and *J*_1800_

As the use of hyperspectral reflectance measurements in high-throughput phenotyping of plant traits continues to increase (Furbank and Tester, 2011), powerful statistical techniques are needed to provide the best predictive power. A common dilemma arises when there are multiple empirical and machine learning algorithms for selection – which one is the best model for high-throughput phenotyping of plant traits (Heckmann et al., 2017)? As the predictive ability of each algorithm may be different, it is worth investigating whether there is a way to collectively harness the strengths of each predictive model. Inspired by the recent advances of geographic stacking in remote sensing applications (Clinton et al., 2015; Healey et al., 2018), this study aimed to test the idea, supported by the results in the sections “The Modeling Performance for Predicting *V*_c,max_ and *J*_1800_” and “The Regression Stacking,” that the stacking of different regression models (ANN, SVM, LASSO, RF, GP, and PLS) would provide a better predictive performance than that of each individual algorithm.

**FIGURE 6:**
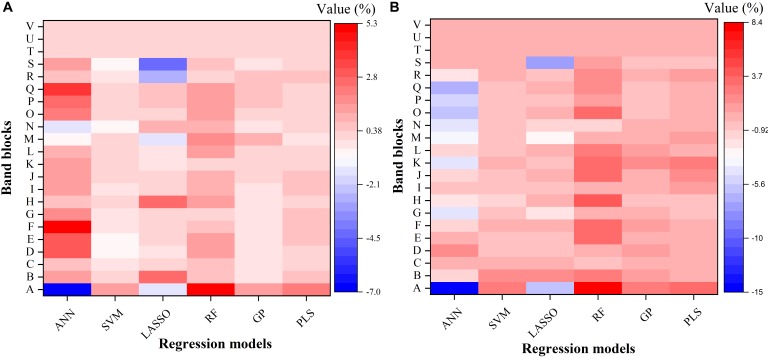
The relative contribution (%) of each band block for the modeling performance of each regression model for estimating *V_c,max_* **(A)** and *J*_1800_ **(B)**. The relative contribution was calculated as the percent change of the R2 value. ANN, artificial neural network; SVM, support vector machine; LASSO, least absolute shrinkage and selection operator; RF, random forest; GP, Gaussian Process; PLS, partial least squares. A: 350–400 nm, B: 400–500 nm, C: 500–600 nm, D: 600–700 nm, E: 700–800 nm, F: 800–900 nm, G: 900–1000 nm, H: 1000–1100 nm, I: 1100–1200 nm, J: 1200–1300 nm, K: 1300–1400 nm, L: 1400–1500 nm, M: 1500–1600 nm, N: 1600–1700 nm, O: 1700–1800 nm, P: 1800–1900 nm, Q: 1900–2000 nm, R: 2000–2100 nm, S: 2100–2200, T: 2200–2300 nm, U: 2300–2400 nm, and V: 2400–2500 nm.

To further understand the modeling performance of each regression technique, the whole spectrum was divided into 22 blocks (A–V in Figure 6). Figure 6 shows the relative contribution (%) of each band block for the modeling performance of each regression technique, including the ANN, SVM, LASSO, RF, GP, and PLS models. For each band block, the modeling procedure was repeated 100 times and the average percent change in the *R*^2^ value was recorded. Here the relative contribution (importance) of each band block to the modeling performance was calculated as the percent change in the *R*^2^ value when the band block was excluded from the modeling procedure. The baseline *R*^2^ value was provided by the model calibrated by the dataset using the whole spectrum from 350 to 2500 nm.

As shown in Figure 6, it was observed that the band blocks T, U, and V (2200–2500 nm) did not affect the predictions of *V_c,max_* and *J*_1800_, as evidenced by the zero percent change of the *R*^2^ values yielded by all the six regression models. This finding suggested that the spectral bands from 2200 to 2500 could be discarded without compromising the overall modeling performance. In addition, the six regression models had quite different responses to the changes in the spectra from 350 to 2200 nm. For the predictions of *V_c,max_*, for example, the exclusion of the band block F (800–900 nm) resulted in the decrease of the *R*^2^ value by 4.9, 0.7, and 0.5% in the ANN, RF, and PLS models, respectively, and the increase of the *R*^2^ value by 0.4 and 0.1% in the SVM and GP models, respectively. For the predictions of *J*_1800_, the exclusion of the band block A (350 – 400 nm) led to a rising *R*^2^ by 14.9 and 5.9 % in the ANN model and the LASSO model, and a falling *R*^2^ by 2.6, 8.4, 2.6, and 3.4% by the other four regression models. Note the percent change value was relatively small for all the six regression models (mostly within the range between -5 and +5), and it should be mainly interpreted as a measure of the relative importance of each band block or the unique contribution of each band block to the modeling performance. For instance, in the SVM model, the percent change value was generally less than 1%, indicating that the unique contribution of each band block was very small, but the shared contribution of the combination of band blocks was huge (99%). The PLS and GP model exhibited a very similar ability as the SVM model to use the shared contribution of the combination of band blocks for the modeling performance. In addition, it should be cautioned that previous studies used the coefficient or the variable importance in projection (VIP) provided by the PLS model to understand the importance of each spectral band to, and the underlying physiological mechanism of the modeling performance (Serbin et al., 2012, 2015; Yendrek et al., 2017). However, when it comes to the comparison of the modeling performance of different regression techniques, these metrics could not be used anymore. Thus, this study used the percent change of the *R*^2^ value as a common metric to understand the modeling differences among the six regression techniques rather than to understand the physiological aspects of correlating reflectance spectra with photosynthetic information. Further explanations of underlying physiology to correlate reflectance spectra with photosynthetic variables can be found in Meacham-Hensold et al. (2019). Overall, the results in Figure 6 suggested that the six regression models utilized information from different spectral regions to achieve the best modeling performance. The differences in utilizing spectral information by the six regression models thus provided a solid basis for stacking which was expected to enhance the strengths of each individual regression technique.

### Implications for High-Throughput Phenotyping

The application of imaging spectroscopy or hyperspectral reflectance to plant phenotyping resulted from initial goals to estimate canopy structure and biochemistry to improve understanding of ecosystem carbon dynamics (e.g., Knyazikhin et al., 2013; Ustin, 2013). Hyperspectral remote/proximal sensing has also been successfully used for rapid measurements of physiological traits in large number of crop genotypes that are needed to fully understand plant-environment interactions (Großkinsky et al., 2015). Previous studies have shown that hyperspectral reflectance measurements and the PLS model can be used together to estimate *V_c,max_* and *J_max_* in a high-throughput manner under well-controlled environment (Serbin et al., 2012; Ainsworth et al., 2014; Silva-Perez et al., 2018). However, the PLS analysis is species and environment dependent and cannot be easily adapted to other crop species with varying field conditions. Inspired by the recent advancements in the geographic stacking in the remote sensing community (Clinton et al., 2015; Healey et al., 2018), this study revealed that the regression stacking was superior over individual regression techniques (ANN, SVM, LASSO, RF, GP, and PLS) in capturing intraspecies variations of photosynthesis capacities among tobacco lines with genetically altered photosynthetic pathways.

The stacking results presented in this study are valuable particularly for high-throughput phenotyping of plant physiology traits of new crop cultivars in a large quantity. Within a field, the microenvironments due to a combination of factors such as temperature, nutrition concentration, and leaf angle distribution may vary from plot to plot and thus influence the plant phenotypes and their interactions with the environment. As a result, spatial and temporal variability of plant traits may be expected due to the variations of microenvironments. The results as shown in Figure 3, 4 indicated that different regression techniques could capture quite different temporal variations of plant photosynthetic capacity. However, variance in *RMSE*/*R*^2^ in the test phase was larger than that in the training phase as shown in Figure 3, 4. This higher variance may suggest that a larger number of data samples are needed to derive a robust statistical relationship between reflectance spectra and photosynthetic variables. Although these machine learning algorithms can still work well with a small number of data pairs, their strength can only be fully released with independent and dependent variables covering a wide range of values. As the collection of ground-truth information is time-consuming, the sharing of photosynthetic variables from different species under different growth environments within the scientific community may be a viable solution to further train and assess each regression technique. The further application of the regression stacking to hyperspectral reflectance measurements from close-range/remote sensing platforms (e.g., unmanned aerial vehicle and gantries) can help estimate photosynthetic capacities of hundreds or even thousands of genotypes needed in a plant breeding context. However, before the use of the developed regression technique at canopy level with close-range/remote sensing platforms, there still exist challenges in detecting continuous variations in photosynthetic capacity among crop cultivars. Leaf-scale analysis provides an ideal test bed for spectroscopic techniques as spectral measurements at a broader scale need to deal with more challenges such as differences in canopy cover and structure among different crop cultivars. Therefore, future work should be made to integrate the developed regression stacking technique with remote sensing radiative transfer models that can accurately estimate reflectance spectra from plants by accounting for canopy structures and background soil signals. Overall, the use of regression stacking yielded a better predictive performance to identify photosynthetic differences among cultivars with a *RMSE* reduction by 8% for *V_c,max_* and by 15% for *J*_1800_.

There is potential for the stacking procedure to be further improved. First, more machine learning algorithms can be incorporated in the stacking procedure. These newly incorporated regression models can be variants of the algorithms already used in the study or totally new machine learning algorithms. For example, deep learning-based regression techniques such as the denoising autoencoder network regression (Bengio et al., 2006) can be used as a totally new algorithm in the stacking procedure while the least square SVM regression (Suykens et al., 2002) can be used as a variant of the SVM regression already used in this study. The inclusion of these different types of regression models may lead to different modeling performance of the stacking. Second, as the stacking procedure occurs at the product level (photosynthesis parameters are separately predicted by each regression technique before stacking), it can be extended to include non-machine learning based approaches. For example, photosynthesis parameters can be estimated by using the ground-based solar-induced florescence (SIF) platform (Grossmann et al., 2018; Yang et al., 2018). The SIF based photosynthetic predictions can then be stacked with those estimated from hyperspectral reflectance to capture interspecies variations among different environmental conditions. Thus, further research efforts can refine this study. It is also worth investigating the portability of the stacking to high-throughput phenotyping of other plant traits such as leaf chlorophyll and nitrogen concentration under varying growth conditions.

## Conclusion

Current efforts to engineer photosynthetic pathways in crops are constrained by phenotyping challenges. Although hyperspectral sensors are increasingly used to rapidly estimate photosynthetic capacity, effective analysis techniques are still lacking to capture interspecies variations in a large field with varying environment conditions. Many machine learning and empirical models can be selected to correlate hyperspectral reflectance with photosynthesis capacity, therefore it is worth investigating which models work better and whether the combination of individual regression techniques can provide better predictive performance. Inspired by the application of geographic stacking in the remote sensing studies, this study examined a series of machine learning algorithms, including ANN, SVM, LASSO, RF, GP, and PLS in the high-throughput phenotyping context. Results showed that the stacked regression had a better predication performance, with an increase of *R*^2^ around 0.1, than individual regression algorithms in phenotyping of photosynthetic capacities. Analysis of variable importance also revealed diverse abilities of the six regression techniques to utilize spectral information for the best modeling performance. The techniques presented in this study could be particularly valuable for high-throughput phenotyping of many crop cultivars, thus accelerating plant breeding processes. It is also suggested in this study that the stacking procedure can be further extended to harness strengths of new techniques such as the ground-based SIF system as a supplement to the hyperspectral reflectance for estimating other phenotypic traits.

## Data Availability

All datasets generated for this study are included in the manuscript.

## Author Contributions

PF and KM-H designed the experiment in consultation with CB and KG. PF performed the data analysis. KG and CB provided advice on data analysis. CB supervised the work as lab leader, advising on experimental design, and data analysis. PF led the development of the manuscript with input from all authors.

## Conflict of Interest Statement

The authors declare that the research was conducted in the absence of any commercial or financial relationships that could be construed as a potential conflict of interest.

## Footnotes

1 http://energyfarm.illinois.edu/index.html
